# The Effect of Coffee and Caffeine Consumption on Patients with Multiple Sclerosis-Related Fatigue

**DOI:** 10.3390/nu12082262

**Published:** 2020-07-28

**Authors:** Lena Herden, Robert Weissert

**Affiliations:** Department of Neurology of the University of Regensburg Hospital, D-93053 Regensburg, Germany; lena.herden@stud.uni-regensburg.de

**Keywords:** coffee, caffeine, multiple sclerosis, fatigue

## Abstract

*Background*: Coffee and caffeine are considered to have beneficial effects in patients with multiple sclerosis (MS), an autoimmune disease of the central nervous system (CNS) that can lead to disability and chronic fatigue. *Methods*: In the present study the preference in terms of coffee and caffeine consumption in patients with MS was assessed. In total the opinions of 124 MS patients were explored with a questionnaire, which was developed to investigate the consumption behavior and associated beneficial and harmful effects of coffee and caffeine concerning symptoms of fatigue. *Results*: Our study showed that 37.1% of the included patients experience severe symptoms of fatigue. In our cohort, fatigue was not related to age, type of diagnosis or duration of the disease. The effects of coffee did not differ between MS patients with and without fatigue. Very few side effects linked to coffee consumption were reported, and we could demonstrate that coffee consumption had no negative impact on quality of sleep. A positive effect on everyday life was observed particularly among patients with a mid-level expanded disability status scale (EDSS). The strongest effects of coffee consumption were observed regarding a better ability to concentrate while fulfilling tasks, an expanded attention span and a better structured daily routine. *Conclusions*: Since coffee showed no severe side effects and in the absence of an effective fatigue therapy, coffee consumption might be a therapeutic approach for selected patients with MS-related fatigue.

## 1. Introduction

Multiple sclerosis (MS) is considered a chronic inflammatory and degenerating disease of the central nervous system (CNS), which often first manifests in early adulthood. What is known so far is that MS is most likely an autoimmune disease with demyelinating processes, which occur in the white and grey matter of the CNS [1]. These lesions, which are visible on nuclear magnetic resonance (NMR) imaging, lead to reduced nerve conductivity in the course of the disease. It is assumed that auto-reactive, myelin-specific T cells are activated in the periphery due to faulty tolerance development. They enter the brain and trigger an immune response by binding to “their” antigen, which leads to an inflammatory process [2]. MS is not a curable disease, but its course can be very positively influenced by medication. MS can manifest in a variety of disease courses. About 85% of patients first develop relapsing-remitting MS (RRMS), which can evolve into secondary progressive MS (SPMS) over a longer period [3]. The other 15% of patients initially develop primary progressive MS (PPMS). The respective diagnosis is made based on the McDonald Criteria [4]. The variety of possible symptoms of MS differs depending on the location and size of the lesions. Common symptoms include visual disturbances, paresis, bladder dysfunction, gait disturbances, as well as paresthesia and hypoesthesia. Another common symptom of MS is fatigue. Fatigue is an extreme exhaustion that usually occurs very suddenly and cannot be compared to being tired [5]. In 2007, the symptoms of patients with MS-related fatigue were examined in detail and a standardized definition was formulated. They summarized that “fatigue is defined as a reversible motor and cognitive impairment, with reduced motivation and the desire to rest. It either occurs spontaneously, or is triggered by mental or physical stress, infection or after eating. Improvement can be achieved by sleeping or resting without sleep. Fatigue can occur at any time but is usually worse in the afternoon. In MS, fatigue symptoms can occur daily, are usually present for years, and are much more severe compared to fatigue caused by other diseases” [6]. More than 70% of people with MS report symptoms of fatigue [7]. Different studies have shown that 14% of patients perceive fatigue as their worst symptom, 55% of patients report it as one of the symptoms that affects them most [8]. Patients suffering from fatigue often do not manage to get through a whole day without taking breaks. As a result, their ability to work is particularly severely affected [5]. Fatigue is also one of the main causes of unemployment or early retirement in people with MS [9,10,11,12]. As therapy, some substances have been tested for their effectiveness. Even a meta-analysis of many pharmacotherapeutic approaches could not define clear therapeutic recommendations [13]. Accordingly, non-drug therapy and comprehensive education about a healthy lifestyle as a therapeutic approach is becoming important, such as the impact of sport and regular physical activity as a preventive measure [13,14]. Therefore, it should be considered whether and to what extent simple therapy approaches, such as coffee or especially caffeine might be an interesting subject for further research. Coffee consists of more than 1000 ingredients, of which caffeine is by far the best studied one. The effect of caffeine is not restricted to a stimulation of the CNS; a short-term improvement of attention, as well as a positive effect on cognition and memory have also been observed [15]. Caffeine reaches its maximum plasma concentration after 20–30 min after intake [16], and caffeine from coffee in particular is absorbed faster as compared to other sources [17]. Due to its hydrophobic structure, caffeine can pass the blood-brain barrier and thus also act on receptors in the brain [16,18]. Its main effect, as a psychostimulant of the CNS, is based on its ability to lower adenosine secretion as an adenosine antagonist on adenosine receptors in certain areas of the brain [19]. Adenosine signals the body that much energy is consumed and causes self-regulation of the body, by having a calming and inhibitory effect via various neurotransmitter-induced pathways [20]. By blocking the adenosine receptors, caffeine prevents adenosine from acting and conversely has a stimulating effect on the CNS. It improves cognitive function, reaction time, concentration, and alertness, as well as motor coordination [21]. The negative reputation of coffee has been reversed in recent years, a coffee consumption of up to four cups per day (a 150 mL, i.e., a total of about 400–500 mg of caffeine) can be considered harmless to human health [22]. Since coffee and caffeine have already shown a positive effect on daytime sleepiness in Parkinson’s disease [23], the question is whether this effect can also alleviate the symptoms of fatigue in MS patients. The connection between caffeine and MS-related fatigue has not been investigated yet. The present study intends to evaluate the possible effect of coffee or caffeine on fatigue as well as on the everyday life of the patients. The aim of this work is to better understand the effect of coffee by means of patient interviews and evaluation of further clinical data. Of interest is the possibility of characterizing a specific group of patients for whom consumption of coffee or caffeine could be indicated as a therapeutic approach.

## 2. Methods

### 2.1. Participants

Questionnaires were distributed to the patients during the weekly consultation hours for MS patients at the Department of Neurology of the University of Regensburg Hospital between March 2018 and September 2018. Inclusion criteria were a confirmed diagnosis of MS and age of majority (18 years). Patients with initial diagnoses, who presented themselves for the first time, were also included. All patients who presented during this period were properly informed about the study and asked to participate. Those who signed the informed consent form were included in the study. The responsible ethics committee of the University of Regensburg approved this retrospective study by data collection in February 2018 (file number 18-890-101).

### 2.2. Data Collection

A short, retrospective questionnaire was prepared to provide an overview of coffee consumption habits in patients with MS. A five-page, simply structured questionnaire was designed, asking about the respective preferences of coffee intake. It focuses on fatigue and behavior concerning coffee consumption. To better classify the patients’ fatigue, we used the Fatigue Severity Score (FSS) [11], as well as the Epworth sleepiness scale (ESS) [24]. Furthermore, patients were specifically asked about problems falling asleep and/or sleeping through the night. The number of hours awake, as well as the frequency of waking up at night were recorded. In addition, the patients were asked to assess whether they felt fit and well rested in the morning or not. Regarding the behavior of coffee consumption, we focused on the reasons why patients do not drink coffee and the possible associated side effects. They were asked what kind of coffee they prefer or which other caffeinated drinks they consume. This, together with the average number of cups consumed per day, as well as the average time of their coffee consumption should provide a better overview. The times of coffee intake were marked on a timeline. Finally, patients should indicate whether, and if so, what subjective effect of coffee they perceive, on what occasions or for what reasons they primarily consumed coffee and what relevance coffee has in their everyday life. Additionally, all patients were neurologically examined and further clinical data, such as the Expanded Disability Status Score (EDSS) [25] were collected. Data about the course of the disease were supplemented from the files.

### 2.3. Data Analysis

Data were coded using SPSS version 25.0.0.1, IBM corporation (Armonk, NY, USA), 2019. Descriptive statistics included the calculation, the distribution, the median and the mean with standard deviation. Differences between groups were presented in cross tables and analyzed by the chi-square-test for categorical variables or t-test for all metric variables. A *p*-value of <0.05 was considered significant. Concerning the presence of fatigue, we decided to set the cut-of score as ≥ four points in the FSS, as originally defined by the authors [26].

## 3. Results

### 3.1. Characteristics of Patients with MS

126 (84.6%) of the 149 questionnaires distributed in the period from March to September 2018 were completed. Two of the 126 questionnaires were filled out incompletely, resulting in a total of 124 (83.2%) completed questionnaires. There was no significant difference between the groups in terms of age, sex, disease duration, diagnosis, or behavior in coffee consumption (Table 1).

Nevertheless, the rate of unemployment was significantly higher in patients experiencing fatigue (*p* < 0.001). In total 34.7% (*n* = 43) were not working anymore at the time of filling out the questionnaire. Out of these 43 patients 67.4% (*n* = 29) stated to not be able to work due to their disease. This was accompanied by an increased EDSS value in the group of patients with fatigue (Table 1).

### 3.2. EDSS

The clinical classification of patients using the standardized EDSS resulted in a median of 2.5 (*n* = 124). For further analysis, we defined three groups based on the EDSS value (Table 2). The EDSS describes the severity of the disease and is correspondingly higher in more severely affected patients. Group-wise a significant difference in mean age could be seen. The duration of the disease since initial diagnosis also showed clear differences in the groups. There was a significant positive correlation between a higher EDSS value and the mean duration of the illness of the patients (r = 0.493, *p* < 0.001). Coffee consumption did not differ significantly between the groups.

### 3.3. Fatigue

As previously described, we formed two groups “fatigue” and “no fatigue”, based on the FSS-Score. In comparison, a higher mean EDSS score in patients affected by fatigue could be shown. The group “fatigue” contained 39.1% with an EDSS score of at least four, in contrast to group “no fatigue” with 19.2% (*p* = 0.003). As described above (Table 1), the groups presented themselves as homogeneous regarding most characteristics. Based on this assumption, we compared the different effects and side effects of coffee and caffeine consumption. As presented in Table 3, the perceived effects of coffee were remarkably similar regarding fatigue.

### 3.4. Sleep Characteristics

In total, 34 of 124 participants (27.4%) stated that they had problems falling asleep. No significant difference in the amount of coffee consumption could be observed in those patients (Table 4). 

**Figure 1:**
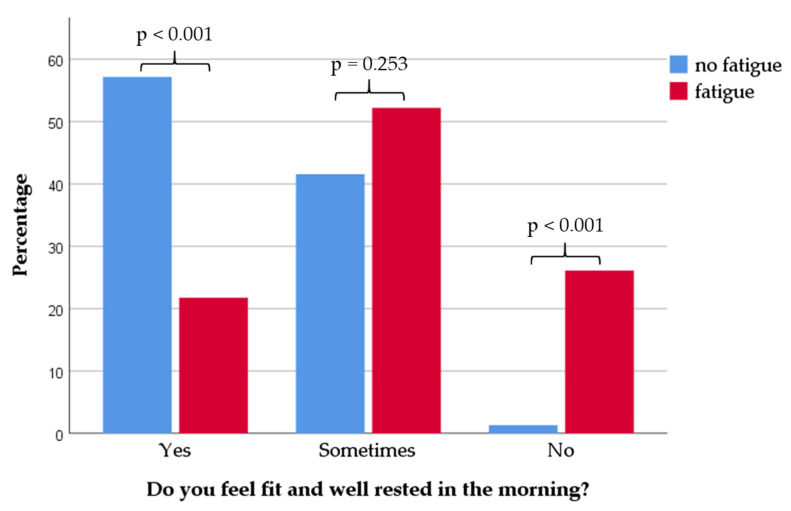
Distribution of sleep quality data in the two groups “fatigue” and “no fatigue”. Patients with fatigue (*n* = 46) stated to feel less active and well rested in the morning (*p* < 0.001), whereas patients without fatigue (*n* = 78) felt more fit in the morning (*p* < 0.001; *p*, statistical significance).

**Figure 2:**
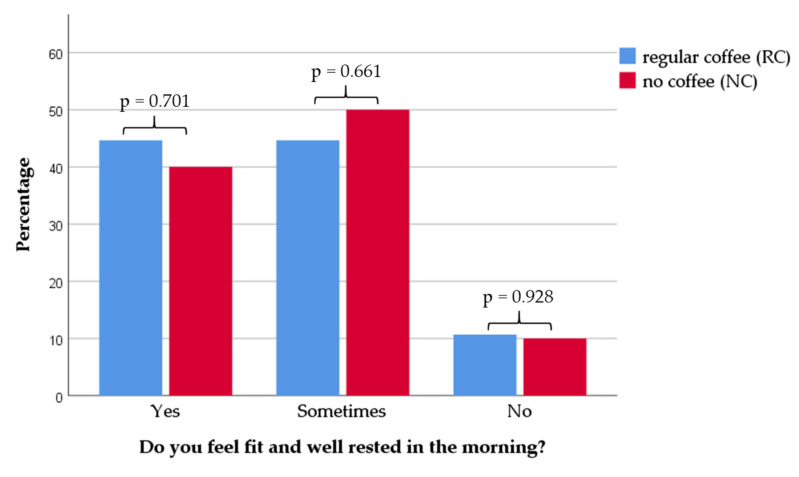
Distribution of sleep quality data in the groups “regular coffee” (RC) and “no coffee” (NC). In the group with RC consumption, patients with an average daily coffee intake of more than 0.5 cups (*n* = 104) are shown. In the group of NC, patients with an average daily coffee intake of lower than 0.5 cups (*n* = 20) are shown. There was no difference in sleep quality regarding coffee consumption (*p*, statistical significance).

Furthermore, 66 of the 124 patients (53.2%) surveyed, said that they regularly woke up more than once during the night. Here as well, no significant correlation with regular coffee consumption could be found. More MS patients laid awake at night who had problems with falling asleep (*p* < 0.001) and had a higher ESS (*p* = 0.013). The frequency of waking up at night was higher in MS patients who had problems with sleeping through the night (*p* < 0.001) (Table 4). Patients with fatigue more frequently replied with “sometimes” or “no” when asked whether they felt fit and well rested in the morning (Figure 1). In contrast no impact of coffee consumption was observed (Figure 2).

### 3.5. Coffee Habits

The amount of coffee consumed per day varied from a minimum of one cup to a maximum of 12 cups (mean = 2.67 ± 2.08) among the patients. For further analysis we built four groups, according to their average coffee intake per day (Table 1). Only fourteen patients stated no coffee consumption at all, most of the patients have reported to consume up to four cups per day.

**Figure 3:**
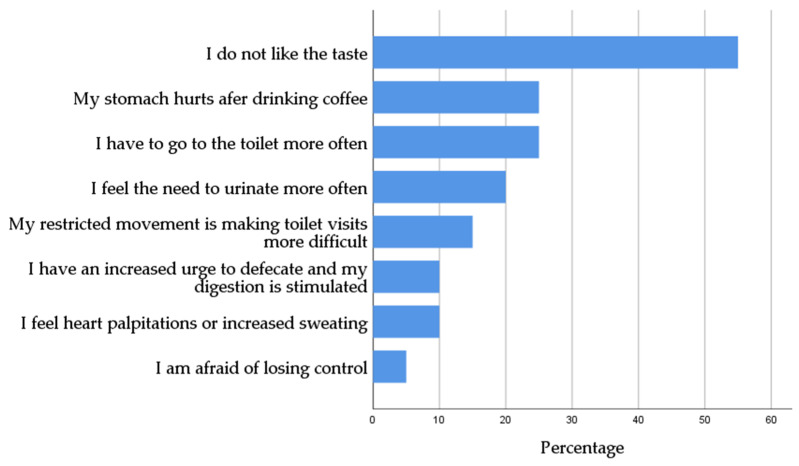
Reasons for patients not to drink coffee indicated in percentages.

The small group of patients with no regular coffee consumption indicated different reasons for this. By far the most prevalent reason for not drinking coffee was a dislike for the taste of coffee (Figure 3).

We evaluated the average time of coffee intake in all participants (Table 5). In total 79.9% of all patients consume their coffee until 6 p.m. Only 8.1% of patients declared to consume coffee after 6 p.m. (7.3% whole day [in the morning, afternoon, and evening]; 0.8% in the morning and evening [until 12 p.m. and after 6 p.m.]). Patients with late coffee consumption showed a higher mean coffee intake of 6.6 ± 2.94 cups per day, compared to 2.3 ± 1.59 cups per day (*p* = 0.001).

The evaluation of a possible correlation between late coffee consumption and the occurrence of sleep problem, showed no significant effect of coffee intake after 6 p.m. (*p* = 0.849).

In the questionnaire, the patients were asked to state their reasons for drinking coffee and the effects they perceived from it. The most frequently selected answer was “I need coffee in the morning so that I can start the day fitter” 46.8% (*n* = 58). While 25.8% (*n* = 32) said that they did not feel any effect from coffee consumption. The effects and side effects did not differ significantly between the groups with different amount of daily coffee intake. The least common reported effect of coffee was stomach problems, such as heartburn (3.2%, *n* = 4). The effects were examined regarding the duration of the illness, for which none of the statements showed a significant difference. Especially the patients of the group “EDSS between 0 and 4” noticed positive effects regarding concentration and attention span (Table 6).

Comparing the four groups with different daily coffee intake there were no significant differences in the EDSS values, or in ESS and FSS values. No significant correlations to age or gender of the patients could be found. No correlation was observed between the amount of coffee intake per day and the presence of a bladder voiding disorder (*p* = 0.514). The information on the current occupation in all groups was like the distribution of the entire patient collective (*p* = 0.205). Furthermore, no differences could be seen in sleep quality or distribution of difficulties falling asleep.

## 4. Discussion

The purpose of this study was to determine the characteristics of patients, for whom coffee consumption might have a beneficial effect on fatigue. This retrospective cohort demonstrated that 46 (37.1%) of the included patients experience severe symptoms of fatigue. The results of this study indicated that fatigue is not related to age, type of diagnosis or duration of the disease. This is different from previous studies in which fatigue was more common in progressive MS forms [27]. Fatigue had a significant impact on the patients’ ability to work with 56.5% of all patients suffering from fatigue stating that they were currently not able to work. 67.4% of these were no longer working due to their disease. This is consistent with previous studies, which identified fatigue as one of the most relevant causes for unemployment in MS [10,12]. Along with this, a significant correlation between a higher EDSS value and a present fatigue could be found.

Looking at sleep quality 27.4% of all patients reported problems with falling asleep. In comparing these patients with those who state no problems, no difference in the behavior of coffee consumption could be found. Even regular late coffee consumption, after 6 p.m. showed no effect on sleep quality and most importantly on the ability to fall asleep. Analyses of the relationship between the ESS value, i.e., the daytime sleepiness and fatigue, showed a positive correlation Other studies found that prevalence of sleeping disorders in MS patients ranged from 25–54% [28,29,30]. Better objective sleep was not related to self-reported scores of sleep-disordered breathing and fatigue [31]. We investigated which criteria had an influence on whether patients felt fit in the morning. Interestingly in our cohort, it could not be observed that coffee consumption had any effect. Furthermore, it could be demonstrated that coffee consumption, regardless of the amount consumed had no negative influence on sleep quality. There was no association either with daytime sleepiness (ESS value) or fatigue (FSS value). This was in contradiction to the frequent assumption that coffee consumption has a negative effect on sleep. Patients with a higher FSS value showed significantly more problems with sleeping.

Coffee and especially caffeine have beneficial effects on various neurological diseases as demonstrated in different studies [32]. Caffeine has also been investigated in in vitro experiments, where it showed a significant positive effect on rodents with experimental autoimmune encephalomyelitis (EAE) [33,34]. In previous research, coffee could lead to various beneficial effects regarding cognition. The overall mood of the patients who consumed coffee was better, and they showed lower fatigue levels. Tiredness and headaches also occurred less frequently among these patients [35].

In this study it was not possible to measure the exact amount of caffeine intake of patients, due to the retrospective nature of the data collection. We evaluated the amount of consumed coffee, measured in cups per day. However, while present studies have shown that an average caffeine content of about 30–175 mg caffeine per cup (defined as 150 mL) can be assumed [36], most studies including a prospective study on the effect of espresso in daytime-sleepiness in patients with Parkinson disease [23] indicate an amount of 90–100 mg per 150 mL. Since the average coffee consumption in our cohort amounted two to three cups per day, an estimated amount of 250 to 300 mg caffeine intake can be presumed. Previous studies demonstrated that an intake of caffeine up to 400 mg can be considered safe and harmless regarding side effects on human health [22]. In total, only 20 of the patients (16.1%) stated not to drink coffee regularly, 14 of them never consume coffee. The reason most indicated for not drinking coffee was simply a dislike for the taste of coffee. The possible perceived side effects tended to play a rather minor role. No differences could be found in terms of sleeping behavior in the small group of patients reporting side effects linked to coffee consumption.

In contrast, significant differences in the perceived effects of coffee consumption depending on disease severity were observed. Especially in patients with an EDSS higher than 0, but below 4, positive effects on everyday life could be identified. These beneficial effects included an increased ability to concentrate for performing tasks, a more focused attention, and a better structure in everyday life. It can be hypothesized that these patients are able to benefit from the effects of coffee consumption due to their still preserved cognitive reserves. The important influence of the cognitive reserve in MS has been demonstrated [37,38,39]. There is also data, which states that this protective role is mostly restricted to memory function and does not refer to the development of the disease in general [40].

Patients with an EDSS score of more than 4 points tended to have a higher quantity of CNS lesions [41,42]. The number of lesions has been shown to be associated with cognitive dysfunction in patients with MS [43]. Lesion load, as defined by conventional NMR imaging techniques, does not correlate with fatigue [44]. However, data show a possible contribution of the gray matter on fatigue development [45]. A recent pilot study demonstrated that patients with MS may link neural resources less efficiently than healthy people, which might result in higher levels of mental fatigue [46]. Even though many studies exist on fatigue and its therapy, treatment options remain extremely limited. The most promising therapy so far has been modafinil. Unfortunately, robust positive effects of modafinil could not be reproducibly shown in every published study [47,48,49]. On the contrary, it has even been shown that physical activity and a well-executed fatigue management in patients with MS have the same, if not a better effect on fatigue than pharmacological therapy [50]. It must be assumed, that various factors, such as diet, activity, and the pharmacological management of MS play an important role regarding fatigue experience [1,51]. 

A limitation of this study is the fact that the sample size is quite small. Possibly, similar studies could be performed with larger numbers of patients with multicenter recruitment. It is further not excluded that the effects of coffee drinking are not necessarily only due to the consumption of caffeine. Coffee contains high concentrations of other potentially bioactive natural products such as trigonelline and chlorogenic acids with partly undefined effects on the human body [32]. In addition, some beverages like tea and soft drinks often contain caffeine which could influence the obtained results to some degree [32].

## 5. Conclusions

The lack of therapeutic options of fatigue in patients with MS is the reason why we initiated this study with the aim to evaluate a simple and maybe helpful approach for fatigue intervention in patients with MS. In our cohort, no negative impact of coffee or caffeine consumption on sleep quality could be found and no serious side effects were observed. Especially MS patients with an EDSS score higher than 0, but lower than 4, noted the strongest effect of coffee consumption on their cognitive abilities, mainly regarding a higher mental capacity and a more structured daily routine.

## Figures and Tables

**Table 1:** Patient characteristics.

		All (*n* = 124)	Fatigue (*n* = 46)	No Fatigue (*n* = 78)	*p*-Value
Age (y)					
	Median (r)	46 (18–80)	49 (20–80)	45 (18–64)	0.116
Sex					
	Female (%)	79 (63.7)	29 (63.0)	50 (64.1)	0.906
	Male (%)	45 (36.3)	17 (37.0)	28 (35.9)	
Diagnosis					
	RRMS (%)	85 (68.5)	29 (63.0)	56 (71.8)	0.177
	PPMS (%)	12 (9.7)	8 (17.4)	4 (5.1)	
	SPMS (%)	14 (11.3)	6 (13.0)	8 (10.3)	
	Initial diagnosis (%)	6 (4.8)	2 (4.3)	5 (6.4)	
	Unspecified (%)	7 (5.6)	1 (2.2)	5 (6.4)	
Duration of disease					
	Mean (y) ± SD	10.45 ± 9.3	11.28 ± 9.4	10.15 ± 9.6	0.532
EDSS (*n* = 120)					
	Median (r)	2.5 (0–8.5)	2.0 (0–8.5)	3.0 (0–8.5)	0.003
Working status					
	Working (%)	81 (65.3)	20 (43.5)	61 (78.2)	<0.001
	Not Working (%)	43 (34.7)	26 (56.5)	17 (21.8)	
Coffee consumption (cups/day)					
	0 cups (%)	14 (11.3)	7 (15.2)	7 (9.0)	0.606
	<2 cups (%)	48 (38.7)	15 (32.6)	33 (42.3)	
	2 to 4 cups (%)	48 (38.7)	19 (41.3)	29 (37.2)	
	>4 cups (%)	14 (11.3)	5 (10.9)	9 (11.5)	

p, statistical significance; n, number; PPMS, primary progressive multiple sclerosis; r, range; RRMS, relapsing remitting multiple sclerosis; SD, standard deviation; SPMS, secondary progressive multiple sclerosis; y, year.

**Table 2:** EDSS distribution of patients with MS.

		Frequency, n (%)	Mean Age (y)	Duration of Disease (y)
Groups (n/%)	EDSS = 0	37 (29.8)	34.8 ± 10.2	5.9 ± 6.1
	EDSS < 4	54 (43.6)	44.7 ± 11.6	9.1 ±7.7
	EDSS ≥ 4	33 (26.6)	53.4 ± 11.5	17.4 ± 11.1
	Total	124 (100.0)	44.1 ± 13.1	10.6 ± 9.5

EDSS, expanded disability status scale, n, number; y, year.

**Table 3:** Effects of coffee consumption of MS patients without and with fatigue.

			No Fatigue (*n* = 78)		Fatigue (*n* = 46)	
“I need the coffee to start the day fitter in the morning”						
	Yes		36 (46.2%)		22 (47.8%)	
	No		42 (53.8%)		24 (52.2%)	
“I am taking deliberate breaks“						
	Yes		37 (47.4%)		19 (41.3%)	
	No		41 (52.6%)		27 (58.7%)	
“I feel more active, so I get a little more exercise in my day”						
	Yes		22 (28.2%)		9 (19.6%)	
	No		56 (71.8%)		37 (80.4%)	
“I have more strength to assert myself in difficult situations and feel more competent in everyday life as well”						
	Yes		4 (5.1%)		4 (8.7%)	
	No		74 (94.9%)		42 (91.3%)	
“I can concentrate better and thus fulfill my tasks”						
	Yes		13 (16.7%)		11 (23.9%)	
	No		65 (83.3%)		35 (76.1%)	
“I can lengthen my attention span and listen more attentively to conversations”						
	Yes		10 (12.8%)		7 (15.2%)	
	No		68 (87.2%)		39 (84.8%)	
“I drink coffee from the “custom” of going out for coffee with someone, e.g., to get to know someone or meet a friend again”						
	Yes		29 (37.2%)		13 (28.3%)	
	No		49 (62.8%)		33 (71.7%)	
“It stimulates my digestion and I notice that I have to go to the toilet more often and more regularly”						
	Yes		22 (28.2%)		11 (23.9%)	
	No		56 (71.8%)		35 (76.1%)	
“I feel my heart beating faster or I’m shaking or sweating afterwards”						
	Yes		3 (3.8%)		4 (8.7%)	
	No		75 (96.2%)		42 (91.3%)	
“I get heartburn or stomachache”						
	Yes		3 (3.8%)		1 (2.2%)	
	No		75 (96.2%)		45 (97.8%)	
“I feel no effect”						
	Yes		21 (26.9%)		11 (23.9%)	
	No		57 (73.1%)		35 (76.1%)	

MS, multiple sclerosis; n, number.

**Table 4:** Characteristics of patients with MS regarding sleep.

	All	Problem	No Problem	*p*-Value
Problems with falling asleep	*n* = 124	*n* = 34	*n* = 90	
Lay awake (h)	0.5 ± 0.94	1.86 ± 0.93	0.4 ± 0.17	<0.001
Coffee consumption (mean in cups)	2.67 ± 2.08	2.98 ± 2.13	2.55 ± 2.06	0.316
ESS (median + range)	7 (0–18)	8.5 (0–18)	6 (0–18)	0.013
Problems with sleeping through the night	*n* = 124	*n* = 66	*n* = 58	
Frequency of waking up (median + range)	1 (0–4)	2 (0–4)	0 (0–1)	<0.001
Regular coffee consumption (%)	83.9	80.3	87.9	0.249
Coffee consumption (mean in cups)	2.67 ± 2.08	3.02 ± 2.33	2.28 ± 1.69	0.051

ESS, Epworth sleepiness scale; h, hours; n, number; p, statistical significance.

**Table 5:** Time of coffee consumption.

Time of Coffee Consumption	*n*	%
In the morning (until 12 p.m.)	24	19.4
In the afternoon (12 p.m. to 6 p.m.)	5	4
In the evening (after 6 p.m.)	0	0
In the morning and afternoon (until 6 p.m.)	70	56.5
In the morning and evening (until 12 p.m. and after 6 p.m.)	1	0.8
Whole day (in the morning, afternoon, and evening)	9	7.3
Never	15	12.0

n, number; p.m., post meridiem (afternoon).

**Table 6:** Effects of coffee consumption categorized based on the EDSS value.

		EDSS = 0 (*n* = 37)	EDSS < 4 (*n* = 54)	EDSS ≥ 4 (*n* = 33)	*p*-Value
“I need the coffee to start the day fitter in the morning”					
	Yes	12 (32.4)	31(57.4%)	15 (45.5%)	0.079
	No	25 (67.6%)	23 (42.6%)	18 (54.5%)	
“I am taking deliberate breaks”					
	Yes	18 (48.6%)	29 (53.7%)	9 (27.3%)	0.049
	No	19 (51.4%)	25 (46.3%)	24 (72.7%)	
“I feel more active, so I get a little more exercise in my day.”					
	Yes	6 (16.2%)	13 (24.1%)	12 (36.4%)	0.151
	No	31 (83.8%)	41 (75.9%)	21 (63.6%)	
“I have more strength to assert myself in difficult situations and feel more competent in everyday life as well”					
	Yes	2 (5.4%)	3 (5.6%)	3 (9.1%)	0.771
	No	35 (94.6%)	51 (94.4%)	30 (90.0%)	
“I can concentrate better and thus fulfill my tasks”					
	Yes	2 (5.4%)	18 (33.3%)	4 (12.1%)	0.002
	No	35 (94.6%)	36 (66.7%)	29 (87.9%)	
“I can lengthen my attention span and listen more attentive in conversations”					
	Yes	3 (8.1%)	13 (24.1%)	1 (3.0%)	0.011
	No	34 (91.9%)	41 (75.9%)	32 (97.0%)	
“I drink coffee from the ‘custom’ of going out for coffee with someone, e.g., to get to know someone or meet a friend again”					
	Yes	12 (32.4%)	18 (33.3%)	12 (36.4%)	0.936
	No	25 (67.6%)	36 (66.7%)	21 (63.6%)	
“It stimulates my digestion and I notice that I have to go to the toilet more often and more regularly”					
	Yes	7 (18.9%)	19 (35.2%)	7 (21.2%)	0.162
	No	30 (81.1%)	35 (64.8%)	26 (78.8%)	
“I feel my heart beating faster or I’m shaking or sweating afterwards”					
	Yes	3 (8.1%)	3 (5.6%)	1 (3.0%)	0.671
	No	34 (91.9%)	51 (94.4%)	32 (97.0%)	
“I get heartburn or stomachache”					
	Yes	3 (8.1%)	0(0.0%)	1 (3.0%)	0,101
	No	34 (91.9%)	54 (100.0%)	32 (97.0%)	
“I feel no effect”					
	Yes	8 (21.6%)	14 (25.9%)	10 (30.3%)	0.661
	No	29 (78.4%)	40 (74.1%)	23 (69.7%)	

EDSS, expanded disability status scale; *n*, number; *p*, statistical significance.
